# Quantitative Susceptibility Mapping and Resting State Network Analyses in Parkinsonian Phenotypes—A Systematic Review of the Literature

**DOI:** 10.3389/fncir.2019.00050

**Published:** 2019-08-06

**Authors:** Esther A. Pelzer, Esther Florin, Alfons Schnitzler

**Affiliations:** ^1^Institute of Clinical Neuroscience and Medical Psychology, Medical Faculty, Heinrich-Heine-University Duesseldorf, Düsseldorf, Germany; ^2^Max-Planck Institute for Metabolism Research, Cologne, Germany

**Keywords:** quantitative susceptibility mapping, magnetoencephallography (MEG), functional magnet resonance imaging (fMRI), motor, non-motor

## Abstract

An imbalance of iron metabolism with consecutive aggregation of α-synuclein and axonal degeneration of neurons has been postulated as the main pathological feature in the development of Parkinson’s disease (PD). Quantitative susceptibility mapping (QSM) is a new imaging technique, which enables to measure structural changes caused by defective iron deposition in parkinsonian brains. Due to its novelty, its potential as a new imaging technique remains elusive for disease-specific characterization of motor and non-motor symptoms (characterizing the individual parkinsonian phenotype). Functional network changes associated with these symptoms are however frequently described for both magnetoencephalography (MEG) and resting state functional magnetic imaging (rs-fMRI). Here, we performed a systematic review of the current literature about QSM imaging, MEG and rs-fMRI in order to collect existing data about structural and functional changes caused by motor and non-motor symptoms in PD. Whereas all three techniques provide an effect in the motor domain, the understanding of network changes caused by non-motor symptoms is much more lacking for MEG and rs-fMRI, and does not yet really exist for QSM imaging. In order to better understand the influence of pathological iron distribution onto the functional outcome, whole-brain QSM analyses should be integrated in functional analyses (especially for the non-motor domain), to enable a proper pathophysiological interpretation of MEG and rs-fMRI network changes in PD. Herewith, a better understanding of the relationship between neuropathological changes, functional network changes and clinical phenotype might become possible.

## Introduction

Quantitative susceptibility mapping (QSM) is a new technique for quantifying magnetic susceptibility (Haacke et al., 2015). It enables quantitative *in vivo* measurement of iron deposition in brain tissue of parkinsonian patients. Technically, QSM solves the deconvolution or inverse problem from a magnetic field to susceptibility source and thus maps the magnetic property of local tissue (de Rochefort et al., 2010). This local property is crucially different from the nonlocal property of traditional gradient echo (GRE) magnetic resonance imaging (MRI), including susceptibility-weighted imaging (SWI), the GRE magnitude T2*-weighted imaging and GRE phase imaging; additionally QSM and GRE MRI are considered to be sensitive to susceptibility (Wang and Liu, 2015). Iron likely stored in ferritin (Griffiths et al., 1999) is highly paramagnetic and can be sensitized in MR imaging by using relaxation contrast [such as T2-weighted imaging and R2 (1/T2) mapping] and susceptibility contrasts [such as T2*-weighted imaging and R2* (1/T2*) mapping; Liu et al., 2015]. R2* mapping has been used for a quantitative study of brain iron (Haacke et al., 2005). A recent postmortem correlation study in seven patients without signs of a neurological disorder has demonstrated that the relationship with R2* can be linear in regions of more uniform iron deposition (Langkammer et al., 2010). However, R2* mapping depends on field strength (Yao et al., 2009), contains substantial blooming artifacts that increase with echo time (Wang et al., 2013), and generally relates to iron concentration in a complex way (Liu et al., 2015). Physically meaningful regularizations, including the Bayesian approach, have been developed recently to enable accurate QSM to study iron distribution, calcification blood degradation, metabolic oxygen consumption, demyelination, and other pathophysiological susceptibility changes, as well as contrast agent bio-distribution in MRI (Wang et al., 2017). This latter development now opened the possibility to apply QSM to study iron distribution in movement disorders like Parkinson’s disease (PD). Due to its novelty, its potential as a new imaging technique for disease-specific prediction of motor (Moustafa et al., 2016) and non-motor symptoms in PD (Chaudhuri et al., 2006) remains elusive. Here, we review the value of QSM imaging in the classification of different parkinsonian phenotypes and the possibility to predict parkinsonian subtypes in combination with well-established functional imaging techniques [resting state functional magnetic imaging (rs-fMRI) and magnetoencephalography (MEG)].

We propose that the combination of structural (QSM) and functional (MEG, rs-fMRI) imaging techniques may enable a more comprehensive view onto PD reflecting structural disease patterns and functional network changes characteristic for motor and non-motor subtypes in PD. These characteristic, disease-related changes measured by the proposed three imaging techniques might help to better classify individual patients with regards to their individual phenotypes. A better classification might then open the possibility for better prediction of disease course and more accurate treatment for the individual patient.

Medical electronic search engine (PubMed) was used to collect studies using QSM, MEG and rs-fMRI in patients suffering from PD (February, 2018). Mainly articles published after the year 2000 were included in the reviewing process; a few important articles, however, date back to the 1990s. The keywords used were: (Parkinson’s disease OR Parkinson disease OR parkinsonian), (quantitative susceptibility mapping OR QSM), (magnetoencephalography OR MEG), (resting state functional magnetic resonance imaging OR functional connectivity in resting state functional magnetic resonance imaging OR resting state MRI). We listed all original studies in English that were retrieved with the search string.

## Quantitative Susceptibility Mapping

Numerous studies questioned if the QSM technique allows measuring the differences in susceptibility in Parkinsonian patients compared to controls. Langkammer et al. (2016) could extend the established finding of higher R2* rates in the SN in PD patients compared to controls by QSM showing superior sensitivity for PD-related tissue changes in nigro-striatal dopaminergic pathways including the pallidum (GP), thalamus (THA), SN and red nucleus (RN). QSM was additionally significantly correlated with the levodopa-equivalent dosage and disease severity. He et al. (2015) compared regional QSM and R2* values in patients with early-stage PD and questioned whether these techniques can be used as biomarkers for early diagnosis in PD. Here, QSM was more sensitive to pathological changes than R2* maps. In QSM measurements, bilateral SN and RN contralateral to the most affected limb showed significantly higher susceptibility values than controls, whereas R2* showed only in the SN contralateral to the most affected limb increased values in PD compared to controls. Furthermore, bilateral SN magnetic susceptibility positively correlated with disease duration and Unified Parkinson disease rating scale (UPDRS)-III scores in early PD. Murakami et al. (2015) also compared R2* measurements and QSM measurements in the following regions: THA, caudate nucleus (CD), GP, SN and RN. They only found significant differences between patients and controls in the SN and again QSM showed higher diagnostic performance than R2*. Barbosa et al. (2015) measured R2, R2* and QSM values in the SN, SNc (compact part), RN, GP, putamen (PUT), THA, white matter and gray matter and only found significant differences in the SN, predominantly in the SNc in comparison of patients with controls; again QSM was the most sensitive quantitative technique to measure iron deposition in the SN. Zhao et al. (2017) also found a significant difference in QSM values in the SN between 29 patients and 25 controls, but differently to the above presented studies, they described no significant differences between patients and controls in the R2* map. As regions of interest (ROIs) they additionally included the RN, GP, PUT and head of CD nucleus without reaching statistical significance. Additionally, they found no correlations of the UPDRS-III score and QSM or R2* values in the Parkinson group. Azuma et al. (2016) performed an ROI analysis in the GP, RN, PUT CD, SN (anterior, medial and posterior part) and analyzed the asymmetry of mean susceptibility in PD compared to healthy controls. They worked out that QSM is useful for assessing the lateral asymmetry and spatial difference of iron deposition in the SN of patients with PD. This spatial difference of iron distribution has been considered in nigrosome-1 imaging, a subregion of the SNc that is specifically altered in PD (Kim et al., 2018). They found that high-spatial-resolution QSM combined with histogram analysis at 3 Tesla MRI could improve the diagnostic accuracy of early-stage idiopathic PD. Du et al. (2016) expanded the analyses to voxel-based midbrain (VBA-) analysis next to the above-described ROI analysis. By the application of midbrain-focused VBA, PD subjects displayed significantly higher QSM and R2* values in the SN. The total volume (cluster size) of significant QSM change was 106 mm^3^ in the right and 164 mm^3^ in the left midbrain, whereas the total volume of significant R2* change was 62 mm^3^, distributed only in the left region of the SNc. In the ROI analysis, the QSM values and R2* values were significantly higher than in the control group with QSM being more sensitive. QSM values additionally correlated with disease duration, levodopa equivalent daily dosage (LEDD) and the UPDRS II. Acosta-Cabronero et al. (2017) performed a different approach. Besides structural analyses (subcortical volumetry, voxel-based morphometry, cortical thickness and tract-based DTI statistics), they performed a whole-brain QSM study to analyze the global distribution of iron accumulation in 25 PD compared to 50 controls. In addition to whole-brain analysis, a regional study including sub-segmentation of the SN into dorsal and ventral regions and qualitative assessment of susceptibility maps in single subjects were also performed. As already presented in the above studies, the most remarkable basal ganglia effect was an apparent magnetic susceptibility increase, which mirrors iron deposition, in the dorsal SN, though an effect was also observed in ventral regions. Increased susceptibility was also found in rostral pontine areas and in a cortical pattern consistent with known PD distributions of α-synuclein pathology: abnormalities were identified in the brainstem comprising the rostral pons (including pyramidal tracts and pontine tegmental areas co-localized with the site of the locus coeruleus), the superior cerebellar peduncle and caudal mesencephalon-seemingly spreading across pars compacta/ventral tegmental SN subregions and midbrain tegmental areas-, possibly also including dorsal raphe and oculomotor nuclei. Parts of the temporal paralimbic, prefrontal and occipito-parietal cortex and, less intensely, insular and cerebellar areas were also involved. In contrast, the striatum, as well as the primary motor and somatosensory fields, were relatively spared. The normally iron-rich cerebellar dentate nucleus (DN) had a susceptibility reduction suggesting a decrease of iron content. No significant changes were found for correlation analysis with the UPDRS-III and Mini Mental Status Examination (MMSE). Guan et al. (2017) analyzed the progressive accumulation of iron in PD patients at different stages of the disease. Forty-five patients with early PD had a Hoehn & Yahr (H&Y) stage of ≤2.5 and 15 patients were defined as late-stage PD with a H&Y stage ≥3. ROI’s were drawn in SN pars reticulata (SNr), SNc, RN, PUT, GP, THA, CD (head) and DN. The SNc showed significantly increased QSM values in the early PD patients compared with the controls. In the late stage of PD regions with increased QSM values extended to the SNr, RN and GP, while the SNc continued to show increased QSM values compared with the controls. Guan et al. (2017) additionally found that the iron content in the SNc and GP was significantly correlated with the H&Y stage, and the SNc was also significantly correlated with the UPDRS-III motor scores. An et al. (2018) subdivided the patients’ group according to clinical scores including the symptom severity (mild, advanced) and parkinsonian subtype [akinetic-rigidic (AR), tremor-dominant (TD) and mixed type (MT)]. In comparison to healthy controls, results redundantly showed significantly increased QSM values in the ROI region SN, whereas patients with more advanced PD showed even higher values. Both subtypes (AR and TD subtype) showed significantly enhanced values compared to controls. The iron content in the SN significantly correlated with the H&Y score, the UPDRS, the Montgomery Asberg Depression Rating Scale (MADRS) and Hamilton Anxiety scale (HAMA). In mildly affected patients, the significant correlation was only with MADRS and HAMA scores. When the disease progressed into advanced severity stage, all these clinical measures (H&Y stage, UPDRS-III, UPDRS total score, HAMA, and MADRS scores) showed a prominent correlation to SN iron content. In the three parkinsonian subtypes (AR, TD, MT), the correlation between iron content and MADRS, HAMA scores was exclusively found in AR subgroups, so that it seems that the AR subgroup was mostly affected by SN iron accumulation.

Next to the analysis of disease-specific Parkinson-related changes and the comparison to healthy controls, Sjöström et al. (2017) retrospectively compared QSM values of 15 patients with progressive supranuclear palsy, 11 multiple system atrophy and 62 PD and 14 healthy controls and found that susceptibility in the RN and GP was higher in progressive supranuclear palsy compared to PD, multiple system atrophy and HC. But susceptibility was higher in multiple system atrophy than in PD and controls and SN susceptibility was increased in PD compared to controls. These findings support that different parkinsonian disorders may have disease-specific topographical patterns of abnormal iron accumulation.

Alkemade et al. (2017) and Liu et al. (2013) could show that the calculation of QSM contrasts contributes to an improved visualization of the entire subthalamic nucleus (STN) and Ide et al. (2015) showed better differentiation between medial and lateral part of the GP and better depiction of medial GP in PD patients. All results are summarized in Supplementary Table S1.

Summing up, four main research questions were part of QSM analyses regarding PD: (1) ROI- analyses of basal ganglia regions (mainly comprising the region of the SN) with correlations to the motor domain; (2) only two studies found correlations with non-motor symptoms (UPDRS-II; MARDS, HAMA); (3) one whole-brain analysis of magnetic susceptibility perturbations in PD exists; (4) QSM is applied for the improved visualization of the STN and the GP.

## Resting State Networks in Magnetoencephalography

MEG is a non-invasive modality for assessing cortical oscillatory activities with high temporal and spatial resolution; it mirrors the summed up excitatory postsynaptic potentials of neuronal populations (Hämäläinen et al., 1993). Neuronal oscillations can be classified into different frequency bands (delta: 1–3 Herz (Hz); theta: 4–7 Hz; alpha: 8–13 Hz; beta: 14–30 Hz; gamma: 30–80 Hz; fast: 80–200 Hz; ultra fast: 200–600 Hz). Functionally, oscillations are features of neuronal activity and the synchronization of oscillations, reflecting temporally precise interaction, is a likely mechanism for neuronal communication (Schnitzler and Gross, 2005). Under normal conditions in healthy subjects, increased synchrony in the beta frequency range accompanies movement preparation and disappears during the actual execution of movements (Schnitzler and Gross, 2005). In PD, synchronized oscillatory activity at beta frequency (13–30 Hz) in the basal ganglia loop is assumed to be anti-kinetic in nature and patho-physiologically relevant for the development of bradykinesia (Brown, 2006). The tremor-related network featuring oscillatory activity is harmonically related to the frequency of the tremor (Timmermann et al., 2003). Treatment with dopaminergic drugs and deep brain stimulation in the STN support the restoration of cortico-cortical interactions at the beta-frequency domain at rest with a reduction in cortical coupling (Silberstein et al., 2005; Hammond et al., 2007).

Regarding actual resting state analyses, some interesting findings have been described that show a slowing of resting state oscillatory activity. Bosboom et al. (2006) elucidated resting state oscillatory brain dynamics *via* MEG in PD patients at moderately advanced stages with and without dementia. They found in a spectral power analysis using Fast Fourier Transformation that in non-demented PD patients, relative theta power was diffusely increased and beta power concomitantly decreased, compared to controls. In central and parietal channels Gamma power was decreased. Dementia was associated with a slowing of resting state brain activity, involving delta and alpha bands, as well as a reduction in reactivity to eye-opening. In *de novo* PD patients compared to healthy controls, Stoffers et al. (2007) could confirm these findings. They applied the synchronization likelihood (SL) method (Montez et al., 2006). This measure is sensitive to both linear and nonlinear interdependencies between signals recorded over distributed brain regions. They further subdivided the alpha band in alpha1 (8–10 Hz) and alpha2 (10–13 Hz). Changes included a widespread increase in theta and low alpha power, as well as a loss of beta power overall but the frontal ROIs and a loss of gamma power overall but the right occipital ROI. Applying again the SL method in MEG, Stoffers et al. (2008a) questioned whether changes in resting-state cortico-cortical FC are a feature of early-stage PD and how functional coupling might evolve over the course of the disease and is related to clinical deficits. They found that drug-naive patients showed an overall increase in alpha1 range compared to controls. With disease progression, neighboring frequency bands were also altered. Alpha2 and beta frequency bands were positively associated with disease duration, whereas the severity of PD was associated with the theta and beta range. They were able to confirm the relationship between beta-band coupling and severity of parkinsonism, specifically bradykinesia, and found some evidence for a similar association between FC in the theta band and motor symptoms, in particular for the symptom tremor. In early-stage PD, cognitive perseveration was positively associated with increased interhemispheric FC in the 8–10 Hz range. They additionally found that the application of dopamine replacement therapy elevated SL in 4–30 Hz range in mild to moderate PD (Stoffers et al., 2008b). A strong motor response was associated with decreases in the local beta-band coupling; these results perfectly complement the findings of Silberstein et al. (2005).

Applying the SL method in MEG, Bosboom et al. (2008) compared 13 demented PD patients with 13 non-demented PD patients. Patients with PD related dementia (PDD) had lower fronto-temporal SL in the alpha range, lower intertemporal SL in delta, theta and alpha1 bands as well as decreased centro-parietal gamma-band synchronization. Moreover, higher parieto-occipital synchronization in the alpha2 and beta bands was found in PDD. Gómez et al. (2011) introduced the Lempel–Ziv complexity (LZC) method. LZC is a complexity measure for finite sequences related to the number of distinct substrings and the rate of their occurrence along the sequence (for further details please see Lempel and Ziv, 1976). They confirmed by this method that PD patients had less complex brain activity in 10 major cortical areas (frontal, central, temporal, parietal and occipital on the right and left hemisphere) and were able to distinguish patients from controls with an accuracy of 81.58%. In line with the above described studies, Pollok et al. (2012) found in an MEG study specifically focussing on S1/M1 region that patients with *de novo* PD have increased synchronized oscillatory activity within sensori-motor loops at beta frequency (13–30 Hz) during resting state modality as well as during isometric contraction compared to controls. The power of the contralateral hemisphere was significantly suppressed during isometric contraction in healthy controls. By contrast, in *de novo* patients both hemispheres were equally strongly activated. In medicated patients, the pattern was found to be reversed with a decrease of synchronized oscillatory activity within sensori-motor loops at beta-frequency. Contralateral beta power was significantly correlated with motor impairment during isometric contraction but not during rest. Pollok et al. (2012) suggested that the reduced ability of the primary motor cortex to disengage from increased synchronized beta-band oscillations during the execution of movements is an early marker of PD.

In a longitudinal study analyzing the cognitive decline in PD, Olde Dubbelink et al. (2013a) found that in contrast to healthy controls, PD patients showed a slowing of the dominant peak frequency. Additionally, analysis per frequency band showed an increase in theta power over time, along with decreases in alpha1 and alpha2 power. In PD patients, a decreasing cognitive performance was associated with increases in delta and theta power, as well as decreases in alpha1, alpha2, and gamma power; increasing motor impairment, however, was associated with a theta power increase only.

A limitation of previous MEG studies (Bosboom et al., 2008; Stoffers et al., 2008a) is that the analyses were performed at the sensor level. Relating the functional sensor-based data to its underlying anatomical substrate is problematic, hampering the interpretation of these results. Additionally, FC calculated on sensor level may result in spurious connectivity due to volume conduction (Schoffelen and Gross, 2009). To overcome this problem, Hillebrand et al. (2012) proposed a new analysis technique, projecting sensor-based data onto an atlas-based source space using beamforming, providing a detailed anatomical mapping of cortical rhythms for 68 regions of interest corresponding to Brodmann areas. Additionally, the phase lag index (PLI) was introduced, because effects of volume conduction can be removed in either signal space or source space by estimating FC using the PLI, which is a measure that quantifies the consistency of non-zero phase differences between two signals (Stam et al., 2007). Olde Dubbelink et al. (2013b) implemented this atlas-based source-space method. To longitudinally investigate resting-state patterns FC in PD patients, eight cortical seed regions were selected: parahippocampal, inferior and middle temporal, temporal pole, orbitofrontal, precuneus, anterior cingulate and middle frontal regions. At baseline, early stage, untreated PD patients (*n* = 12) had lower parahippocampal and temporal delta band connectivity and higher temporal alpha1 band connectivity compared to controls. In a larger patient group (*n* = 43), longitudinal analyses over a 4-year period uncovered decreases in alpha1 and alpha2 band connectivity for multiple seed regions that were associated with motor or cognitive decline (alpha1: middle temporal cortex; alpha2: parahippocampal, inferior temporal, middle temporal, precuneus). Ponsen et al. (2013) applied the same method and compared 13 patients with PDD with 13 non-demented PD patients. Compared to PD patients, PDD patients had more delta and theta power in parieto-occipital and fronto-parietal areas. The PDD patients had less alpha and beta power in frontal and parieto-temporo-occipital areas. PDD patients had lower mean PLI values in the delta and alpha bands in fronto-temporal and parieto-temporo-occipital areas when compared to PD patients. Additionally, in PDD patients, connectivity between pairs of regions of interest (Brodmann areas) was stronger in the theta band and weaker in the delta, alpha and beta bands.

In order to examine large-scale structures of resting-state brain networks in PD, using concepts from graph theory, Olde Dubbelink et al. (2014) applied MEG measurements in 70 PD patients and 21 healthy controls in a longitudinal analysis over a 4-year period. They showed that impaired local efficiency (i.e., local clustering of connections) and network decentralization are very early features of PD that continue to progress over time, together with reduction of global efficiency (i.e., long-distance connections). By combining resting state MEG measurements with cognitive assessments, Olde Dubbelink et al. (2014) was able to predict dementia in PD better than with one factor alone (hazard factor: 27, 3; *p* < 0.001). Heinrichs-Graham et al. (2014) examined resting-state neurophysiological activity before and after dopamine replacement therapy in the motor network of patients with PD; they then compared this data to a group of matched controls without neurological diseases. They found that untreated patients with PD exhibited significantly decreased beta oscillations compared to controls in the bilateral motor regions and that this difference is almost normalized following dopamine replacement. Despite decreased beta oscillatory activity in the primary cortices, they found that patients with PD exhibited higher synchronicity between the left and right motor cortex, which was limited to the beta frequency and was partially normalized by dopamine replacement therapy. Boon et al. (2017) analyzed *via* MEG the pathophysiological mechanisms underlying PD cognitive decline and conversion to PD dementia. Preferential beta band information outflow was significantly higher in PD patients compared to controls for the basal ganglia and fronto-temporal cortical regions, and significantly lower for parieto-occipital regions. Additionally, in patients, low information outflow from occipital regions correlated with poor global cognitive performance. Boon et al. (2017) introduced the hypothesis that in the PD brain, a shift in balance towards a more anterior-to-posterior beta band information flow takes place and is associated with poorer cognitive performance.

But also in DBS resting state MEG becomes more and more applicable. Cao et al. (2015) investigated the effect of STN-DBS on spontaneous cortical oscillations of patients with PD, which were detected non-invasively with whole head MEG. Without surgery, a prominent slowing of resting oscillatory activities compared with healthy controls was found, consistently with previous studies (Bosboom et al., 2006). With STN-DBS switched-on, an improvement of the PD symptoms was found by suppressing the synchronization of alpha rhythm in the somato-motor region. Oswal et al. (2016) performed simultaneously MEG and intracranial local field potential (LFP) recordings and compared differences in STN-cortical coherence topographies at rest and during clinically effective high-frequency stimulation of the STN. Within the STN, DBS suppressed synchronized neuronal activity, preferentially at low beta (13–21 Hz) rather than high beta frequencies (21–30 Hz). Suppression in the low beta band correlated with motor improvement. In contrast, DBS suppressed the coupling of STN to cortical motor regions across the whole beta frequency band; this did not correlate with clinical improvement. The effect of STN DBS on coupling with cortex was spatially selective and restricted to mesial cortical areas, reflecting that coupling mediated predominantly by the hyperdirect and indirect pathways to STN (Alexander and Crutcher, 1990; Nambu et al., 2002). All results are summarized in Supplementary Table S2.

Summing up, three key findings from the MEG literature should be mentioned: (1) In PD, we can find a slowing of resting state oscillatory activity compared to controls, which increases during disease progression and is especially enhanced during cognitive decline. (2) The increased synchronization of beta-band activity is a pathognomic feature for the deficient motor action in PD and administration of dopamine decreases beta-band synchronicity with an improvement of motor output. (3) Methodologically, a combination of whole-head-MEG and basal ganglia LFP recordings are nowadays possible.

## Resting State Networks in Functional Magnetic Resonance Imaging

Spontaneous fluctuations in brain activity, as they have been described for rs-fMRI, have been detected *via* functional MRI (Fox and Raichle, 2007). The blood oxygen level dependent signal (BOLD) is an indirect measure of changes in neuronal activity. Examinations of rs-fMRI provide a non-invasive method that focuses on low-frequency spontaneous fluctuations (i.e., below 0.1 Hz) in the BOLD signal, which occurs when individuals are at rest. Numerous methods have been developed and used to study resting state FC like: (1) seed-based FC; (2) hierarchical clustering; (3) graph theory; (4) independent component analysis (ICA); (5) regional homogeneity (ReHo); (6) amplitude of low frequency fluctuation (ALFF); (7) Granger causality analysis (GCA; for a detailed overview see e.g., Prodoehl et al., 2014). A number of studies have consistently reported the formation of functionally linked resting-state networks during rest; these studies—although using different groups of subjects, different methods (e.g., seed-based FC, ICA and others) and different types of MRI protocols—, showed a large overlap between their results and indicated the robust formation of functionally linked resting state networks in the brain during rest (van den Heuvel and Hulshoff Pol, 2010).

In PD, changes of FC have been reported for different networks. We revise the literature of the most frequent networks: (1) a primary somato-motor network; (2) default mode network (DMN); (3) the left and right parietal-frontal network; (4) the salience network; and (5) a primary visual area and extra-striate visual network.

### The Somato-Motor Network

Specific changes in functional neuroimaging for the sensori-motor network of patients with PD have been recently found (Tessitore et al., 2014). Wu et al. (2009) applied graph theory to examine the resting motor network in patients with mild to moderate PD, testing both with and no medication. Patients also performed a finger-tapping task to identify ROIs for the resting state motor network analysis. They found that PD patients after medication withdrawal had significantly decreased FC in supplementary motor area (SMA), left dorsal lateral prefrontal cortex (DLPFC) and left PUT and increased FC in the left cerebellum, left primary motor cortex and left parietal cortex compared to healthy controls. Application of levodopa relatively normalized the pattern of FC in PD patients. This normalization of SMA FC after levodopa administration has also been confirmed by a recent study of Esposito et al. (2013), combining an FC analysis and a spectral frequency analysis. Levodopa stimulated reduced signal fluctuations in the SMA with a selective frequency band of the sensorimotor network. Under dopaminergic therapy, Göttlich et al. (2013) even found increased connectivity within the sensorimotor network and parietal areas besides decreased connectivity in the CD nucleus, orbitofrontal and occipital regions. In another study, Wu et al. (2011) analyzed PD patients after >12 h withdrawal of levodopa compared to healthy controls *via* FC analysis. They investigated the interplay between the rostral SMA (also known as pre-SMA; mainly involved in motor preparation and initiation) and the primary motor cortex (M1; mainly involved in motor execution) by choosing these two regions as seed regions. Without dopaminergic medication, connectivity within the pre-SMA in patients with PD compared to healthy controls was increased to the right M1 and decreased to the left PUT, right insula, right premotor cortex and left inferior parietal lobule. Stronger connectivity was only found in M1 within its own local region in PD patients compared to controls. In contrast to these findings, Yu et al. (2013) found enhanced connectivity between the PUT and the SMA, although patients were also withdrawn >12 h from dopaminergic medication.

But functional changes have been found not only in cortical regions. Helmich et al. (2010) for example compared the FC profile of the posterior PUT, the anterior PUT and the CD nucleus between 41 patients and 36 matched controls and found that the posterior PUT was uniquely coupled to cortical motor areas (like SMA and M1), the anterior PUT to the pre-SMA and anterior cingulate cortex and the CD nucleus to the DLPFC. PD patients had decreased connectivity between the posterior PUT and the cortex (bilateral M1 and secondary somatosensory cortex, intra-parietal cortex, insula and cingulate motor area) after 12 h dopaminergic withdrawal. Differences between PD and healthy controls were, however, specific to the PUT: although PD patients showed decreased coupling between posterior PUT and the inferior parietal cortex, this region showed increased FC with the anterior PUT, suggesting a (maybe compensatory) modulation of existing functional networks.

In light of the study design of Helmich et al. (2010), Manza et al. (2016) also subdivided the striatum in an anterior, posterior PUT and ventral and posterior CD nucleus in early-stage PD. First, they found that higher motor deficit rating was associated with a weaker coupling between the anterior PUT and midbrain including SN; second, a decline in cognitive function, particularly in the memory and visuo-spatial domains, was associated with stronger coupling between dorsal CD nucleus and rostral anterior cingulate cortex. Following Braak’s theory (Braak et al., 2004), a recent study by Hacker et al. (2012) focussed on changes in functional network integrity regarding the brainstem. They compared 13 patients with PD and 19 age-matched controls and found that the PD group had markedly lower striatal correlations with the THA, midbrain, pons and cerebellum. Comparing PD to controls, focally altered FC was also observed in sensori-motor and visual areas of the cerebral cortex, as well as in the supramarginal gyrus.

By applying a seed-based approach with four ROI’s in each hemisphere (CD, PUT, GP and THA), Agosta et al. (2014) compared cortico-striatal-thalamic network FC in treated and untreated PD patients and controls to study the effect of levodopa on these networks. Patients without dopaminergic stimulation had an increased FC between the left and right basal ganglia and decreased connectivity of the affected CD nucleus and THA with ipsilateral frontal and insular cortices. Compared with healthy controls and untreated PD patients, PD patients under dopaminergic stimulation showed a decreased FC among the striatal and thalamic regions and increased FC between the striatum and temporal cortex and between the THA and several sensori-motor, parietal and occipital regions. Summing up, levodopa in this study was able to facilitate a compensation of functional abnormalities through an increased FC in the THA. In both groups (untreated/treated), patients with a more severe motor disability had an increased striatal and/or thalamic FC with temporal, parietal, occipital and cerebellar regions.

Sharman et al. (2013) found in general reduced sensori-motor circuit connections within the basal ganglia and between basal ganglia and the THA in PD patients without dopaminergic medication compared to healthy controls. The sensori-motor cortex showed reduced connectivity with the THA. The globus pallidus showed reduced connectivity with both the PUT and the THA in PD patients. Connections of the SN were reduced with the globus pallidus, the PUT and THA in PD patients compared to controls. Increased FC was only observed for non-sensori-motor connections between PUT and associative cortex, THA and limbic cortex as well as between the PUT and THA.

Bell et al. (2015) also found impaired striatal interconnectivity in PD without dopaminergic medication with a pathological decoupling of the striatum from the thalamic and sensori-motor networks. The application of dopaminergic medication significantly improved connectivity across striatal subdivisions.

Kwak et al. (2010) applied FC and frequency content analysis to study the modulation in basal ganglia thalamo-cortical networks in six striatal seed regions [(1) inferior; (2) superior ventral striatum; (3) dorsal CD; (4) dorsal caudal; (5) rostral; and (6) ventral rostral PUT]. In apparent contradiction with the results of Kwak et al. (2010), Esposito et al. (2013) reported that levodopa treatments reduce, rather than increase, the amplitude of low-frequency oscillations, thereby restoring the normal oscillations in an otherwise functionally hyperconnected resting brain. Both studies have, however, remarkable methodological differences (drug-naïve PD vs. levodopa withdrawal in chronically treated PD patients; ICA vs. “full variance” voxel-level time course), so these results should be interpreted carefully.

By studying the sensori-motor system, one important nucleus should not be neglected: the STN. Baudrexel et al. (2011) investigated alterations in the FC profile of the STN in a voxel-by-voxel fMRI study by comparing early-stage PD patients (*n* = 31) after dopaminergic withdrawal with healthy controls (*n* = 44). The analysis revealed increased FC between the STN and cortical motor areas [primary motor cortex (M1), premotor cortex and SMA] in line with electrophysiological studies. FC analysis in the M1 hand area elucidated that the FC increase was primarily found in the STN area within the BG, suggesting that increased STN-motor cortex synchronicity mediated *via* the so-called hyperdirect motor cortex-subthalamic pathway might play a crucial role in the pathophysiology of PD. Increased FC between these two areas was also found in early drug-naïve PD patients before application of dopamine (Kurani et al., 2015). Few articles reported FC of STN and motor area in PD patients during the intake of dopaminergic medication. Fernández-Seara et al. (2015) showed an increased FC between the STN and motor cortex just like in PD patients after dopaminergic medication by using arterial spin-labeled perfusion fMRI, whereas Mathys et al. (2016) did not find a change in the FC between the two areas. Shen et al. (2017) compared changes in 31 PD patients under dopaminergic medication with healthy controls and found that, in line with the findings of Braudexel, an increased FC was found between the STN and the sensorimotor cortex, which was related to motor symptom severity in on-medicated patients. All results are summarized in Supplementary Table S3.

### The Default Mode Network

The DMN consists of the following brain regions: the precuneus, medial frontal, inferior parietal cortical regions and medial temporal lobe. Studies analyzing changes in the DMN in PD have produced diverse results. Applying ICA, Krajcovicova et al. (2012) found no differences in the DMN between patients and controls, comparing cognitively intact patients and controls; patients were however on dopaminergic medication and were not subdivided by the different phenotypes (AR; TD). Comparing PD cognitively unimpaired patients and controls by applying ICA *via* resting state MRI, FC have been found to be decreased in the right medial temporal lobe and bilateral inferior parietal cortex within the DMN (Tessitore et al., 2012b). Karunanayaka et al. (2016) again applied ICA and questioned whether PD_AR_ has different FC patterns in the DMN when compared to PD_TD_ and healthy controls; they found that there was a decreased activity in the left inferior parietal cortex and the left posterior cingulate cortex. PD patients were treated with anti-parkinsonian medication, except for two subjects, who had very mild symptoms and were drug-naïve. Due to the fact that dopamine replacement therapy has an influence on resting state networks (Tahmasian et al., 2015), Hou et al. (2017) included cognitively unimpaired and drug-naïve PD_AR_ patients in his study. Still, they found a decline in FC of the posterior DMN and enhanced compensatory FC of the anterior DMN in early-stage drug-naïve PD_AR_ prior to clinical evidence of cognitive impairment. Non-demented PD patients with and without hallucinations showed reduced connectivity during resting state in the DMN compared to healthy controls. Patients with hallucinations had however significantly greater connectivity in the DMN compared to patients without visual hallucinations. The levodopa dosage was controlled for both groups (Yao et al., 2014).

Disbrow et al. (2014) found a dependency between FC in the DMN and the cognitive dysfunction in non-demented PD patients compared to healthy controls. They found that DMN FC was decreased in the PD group, specifically between posterior cingulate, medial prefrontal and inferior parietal nodes. Greater DMN-FC was related to faster processing speed in the PD group.

Amboni et al. (2014) went one step further and investigated whether patients with mild cognitive impairment (MCI) have differences in FC in the DMN compared to patients without MCI. Both groups were then further compared to healthy controls. Again, both PD groups showed a general decrease in the DMN connectivity compared with controls. But PD patients with MCI additionally showed a decreased FC of the bilateral prefrontal cortex within the fronto-parietal network. The decreased prefrontal cortex connectivity correlated with cognitive parameters, but not with clinical variables. Hou et al. (2016) compared drug-naïve patients with MCI with patients with unimpaired cognition and healthy controls in a seed-based approach. They also found reduced FC in the DMN in patients with MCI compared to healthy controls, but also in a set of other regions including the precentral gyrus, middle temporal gyrus, insula, anterior inferior parietal lobule and middle frontal gyrus. In the DMN patients with MCI had decreased FC between the hippocampal formation and inferior frontal gyrus, between the posterior cingulate gyrus and posterior inferior parietal lobule and between the anterior temporal lobe and inferior frontal gyrus compared to controls. In another seed-based approach, Gorges et al. (2015) compared the intrinsic FC in cognitively-unimpaired and cognitively-impaired patients. They found that cognitively- impaired patients compared to healthy controls mainly had decreased FC in the DMN. Patients who were cognitively unimpaired had network expansions with significantly increased intrinsic FC in cortical, limbic and basal-ganglia-thalamic areas; as suggested by the authors this might be an adaptive (compensatory mechanism) by recruiting additional resources to maintain normal cognitive performance. Changes in the DMN associated with cognitive impairment have also been confirmed by a recent study of Lucas-Jiménez et al. (2016). Changes of FC in the DMN have also been related to saccadic performance in PD (Gorges et al., 2013); changes in saccadic performance are described to be associated with cognitive changes (Mosimann et al., 2005).

The interaction of the DMN with other structures in the brain also needs to be considered. Hu et al. (2015), for example, compared 20 depressed with 40 non-depressed PD patients and 43 controls. They found stronger connectivity between the left median cingulate cortex and the DMN in depressed PD patients.

### The Fronto-Parietal Network

*Via* functional activation studies it has been shown that the fronto-parietal network, which consists of the DLPFC and the posterior parietal cortex (PPC) is involved in the “top down” control of executive control (Markett et al., 2014; Gratwicke et al., 2015). Tessitore et al. (2012a) reported in a fMRI resting state paradigm that patients with freezing of gait (FOG) had a different pattern of activation in the executive attentional network e.g., the right fronto-parietal network (right middle frontal gyrus) as well as in visual networks (right occipito-temporal gyrus). Their findings suggested that a disruption of “executive-attention” and visual neural networks is associated with the development of FOG. Regarding the amplitude of low-frequency fluctuation (ALFF), Zang et al. (2007) hypothesized that ALFF measures the amplitude of low-frequency (0.01–0.08 Hz) BOLD signal and can be used as an index of local spontaneous neural activity in resting state. Mi et al. (2017) applied this resting state method in PD patients with FOG and compared resulting ALFF measures to patients without FOG and HC. They found that FOG is associated with a dysfunction within fronto-parietal regions, along with increased inhibitory output from the basal ganglia. Additionally, they reported altered activity of cerebellum, implicating its role in the pathophysiology of FOG. Comparing patients with postural instability and gate disorder to TD patients, the latter group had specific hyper-connectivity between motor cortical areas and the inferior parietal lobule. These findings, correlated with a reduced behavioral impairment, suggest compensatory mechanisms in PD patients with tremor (Vervoort et al., 2016). FOG is frequently associated with depression (Giladi and Hausdorff, 2006) and interestingly also depressive PD patients show increased FC in the left fronto-parietal network in addition to increased FC in basal ganglia and decreased FC in salience network and DMN (Wei et al., 2017). The authors also reported hyper-connectivity between the DMN and the left fronto-parietal network in depressed PD. Boord et al. (2017) examined task activated attentional networks and their possible relationship with FC changes in resting state; they found that a weakened interaction between the default mode and task-positive networks might alter the way in which the executive response is processed in PD. Higher activation was found in patients with PD in four regions of the dorsal attention and fronto-parietal networks, namely right frontal eye field, left and right intraparietal sulcus, and precuneus during increased executive challenges. In three regions, they worked out reduced resting state connectivity to the DMN. Further, whereas higher task activation in the right intraparietal sulcus correlated with reduced resting state connectivity between right intraparietal sulcus and the precuneus in healthy controls, this relationship was absent in PD subjects.

### The Salience Network

The salience network is an intrinsically connected large-scale network anchored in the anterior insula (AI) and dorsal anterior cingulate cortex (dACC; Menon and Uddin, 2010). Next to the AI and the dACC, it includes three key subcortical structures: the amygdala, the ventral striatum and the SN/ventral tegmental area. In the past, the insula was thought to be primarily a limbic cortical structure. Nowadays, it has become clearer that this part of the brain is highly involved in integrating somatosensory, autonomic and cognitive-affective information to guide behavior. Thus, it acts as a central hub for processing relevant information related to the state of the body as well as cognitive and mood states. According to Braak’s staging hypothesis of PD progression, alpha-synuclein is highly deposited in the insula when Braak’s stage 5 is reached (Braak et al., 2006). An involvement of the insula in the generation of non-motor symptoms in PD seems likely (Christopher et al., 2014).

Nowadays, apart from pure task-related activations in fMRI, resting state analysis allows the measurement of FC in the salience network at rest. Wu et al. (2011) for example found that the right mid-AI has reduced FC to the pre-SMA in PD compared with healthy controls (Wu et al., 2011). In addition to motor symptoms, non-motor symptoms, as they are frequently present in PD, should also be considered: fatigue might be explained by a dysfunction of the salience network (Li et al., 2017; Zhang et al., 2017). But also a role in the cognitive decline in PD has been found for the salience network. Especially the coordination of switching between different networks (like DMN, central executive or dorsal attention network) in cognitive tasks seems to be regulated by e.g., the AI and the ACC (Sridharan et al., 2008; Baggio et al., 2015). The central executive network (CEN) is a fronto-parietal network that is crucial to working memory and cognitive control of thought, emotion and behavior (Menon, 2011). Regarding impulsive control behaviors (ICB), the presence of ICB symptoms was associated with increased connectivity in the salience network and the DMN, as well as with decreased connectivity within the CEN (Tessitore et al., 2017b). Drug-naïve PD patients who develop ICB in a 36 months observation time period showed at baseline a decreased connectivity in the DMN and CEN and increased connectivity in the salience network. These results suggest that these cognitive and limbic connectivity changes are predictive for the development of ICB in PD (Tessitore et al., 2017a).

### Visual Network

Visual hallucinations are the most common manifestation of psychosis in PD and are predictive of a rapid cognitive decline. Rektorova et al. (2012) compared patients with Parkinson’s disease dementia (PDD) with patients without dementia and controls *via* seed-based fMRI. Using the Cd nucleus as a seed for the extra-striatal visual resting state network, they found significant decreases of connectivity in the left and right inferior occipital gyrus in PDD as compared to HC, in addition to connectivity changes within the DMN.

Summing up, in rs-MRI analyses, patients with PD present with multiple network changes like: (1) abnormal signaling in the SMA; (2) decreased functional connectivity between the striatum and cortical regions; (3) abnormal STN-motor-cortex synchronicity resulting in motor impairment, like bradykinesia. Functional connectivity changes in the (4) DMN are frequently found not only in cognitively impaired PD patients, but also in patients with FOG and depression. Fatigue, but also cognitive decline, are caused by impaired functional connectivity of the; (5) salience network. In addition to connectivity changes within the DMN, significant decreases of connectivity; and (6) in the left and right inferior occipital gyrus appear in patients with PDD (Rektorova et al., 2012).

### Limitations of Functional rs-fMRI

It is important to note that we only referred to articles about “functional connectivity” (FC) in this review article. FC only refers to the statistical interdependence of the signal from different areas (for an overview see e.g., Friston, 2011). In addition, other possibilities to measure connectivity exist in neuroimaging: (i) structural connectivity or so-called anatomical connectivity, which refers to the existence and structural integrity of tracts connecting different brain areas (Jbabdi et al., 2015); (ii) effective connectivity, which brings the element of causation into the connection analysis, that means that activity in one area causally affects activity in another area (for further details see Friston, 2011).

The rs-fMRI for functional connectome, to which we referred to in this review, has obvious limitations, as it is neither able to give information about the underlying structure nor the direction and how different areas are influenced by each other. Therefore, our proposal of combining different methodological approaches (QSM, rs-fMRI, MEG) should be seen as an idea/starting point but does still not tell the whole truth of the underlying network. The interpretation of combinations between the different connectome analyses are challenging and are part of actual discussions, see e.g., Uddin (2013), Wang and Liu (2015) and Stam et al. (2016). To characterize brain networks properly, structural and effective connectivity analyses should also be part of combination studies in order to adequately elucidate pathological network changes in e.g., neurodegenerative diseases to classify parkinsonian phenotypes.

## Discussion

In this review article, we present three different imaging entities, which enable a view into structural and functional abnormalities in PD patients: (1) QSM imaging; (2) MEG; and (3) rs-fMRI. All described methods have a high potential to analyze pathophysiological changes in PD networks with a different accentuation of network changes in both the motor and non-motor domain, as summed up exemplary in Supplementary Tables S1–S3.

As can be seen in the different tables, the most challenging factor for the prediction of the parkinsonian phenotype is a proper control of the heterogeneity of parkinsonian symptoms. In all studies we found: (i) PD-independent factors (e.g., age, handedness, gender); (ii) PD-characteristic factors like the existence of (a) non-motor symptoms (cognitive functioning, obstipation, hallucinations e.g. Wu et al., 2017); (b) motor symptoms (tremor, rigor, akinesia, postural instability e.g., Obeso et al., 2017). Additionally, (c) symptom onset; (d) disease duration; and (e) duration of dopaminergic treatment with dopaminergic agonists or levodopa and the (f) treatment response to dopamine were important to characterize the individual PD phenotype. All these symptoms and characteristics can appear simultaneously in one patient; but some patients solely have one single symptom. This heterogeneity makes a prediction of a single course of the disease challenging and methods are needed to better understand the pathophysiological background of differences in the development of the clinical phenotype. This knowledge could help to potentially modulate the disease course of each patient individually.

**Figure 1:**
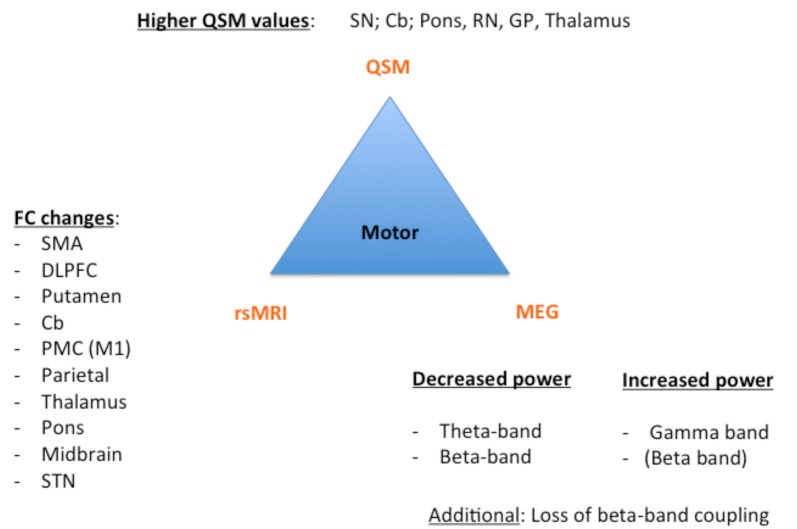
Key results of the motor phenotype. For all three techniques [Quantitative susceptibility mapping (QSM), resting state magnetic resonance imaging (rsMRI), and magnetoencephalography (MEG)], we summarized the most important regions and frequency changes typical for the individual phenotype. FC, functional connectivity; SMA, supplementary motor area; DLPFC, dorsolateral prefrontal cortex; Cb, cerebellum; PMC, premotor cortex; STN, subthalamic nucleus; SN: substantia nigra; RN, red nucleus; GP, globus pallidus.

**Figure 2:**
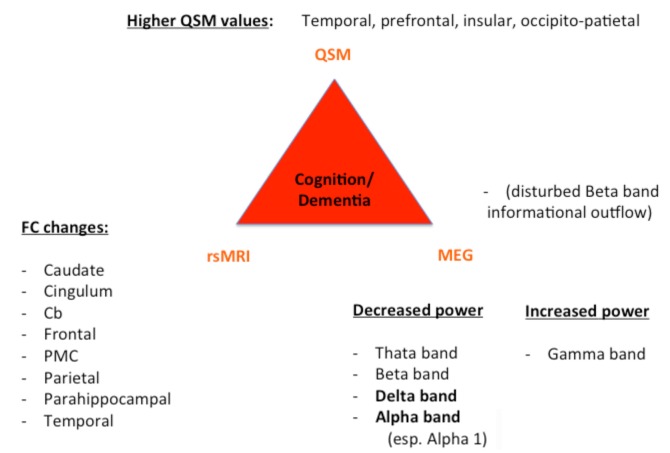
Key results of patients with cognitive decline/dementia. For all three techniques (QSM, rsMRI, and MEG), we summarized the most important regions and frequency changes typical for the individual phenotype. FC, functional connectivity; Cb, cerebellum; PMC, premotor cortex.

Despite these challenges, a combination of structural (QSM) and functional data (rs-MRI, MEG) might allow a further classification and a possible prediction of the parkinsonian phenotypes as exemplarily proposed for the impaired motor network in Figure 1 and cognitively-impaired/demented patients in Figure 2: (1) whereas the key knowledge in QSM imaging in PD lies at the actual time point in the ROI-analysis at subcortical level (specifically in the SN) and correlation analyses with disease-specific changes regarding the motor domain; (2) MEG studies predominantly investigate the influence of network changes explaining motor symptoms (e.g., *via* changes in the beta band) and non-motor symptoms (e.g., *via* the increase of slowing of brain activity specifically in demented PD patients) at cortical level. The whole-brain analyses of (3) rs-fMRI build a bridge between QSM imaging and MEG to combine subcortical and cortical network changes. Rs-fMRI, as already mentioned above, has a high impact to improve our understanding of global network changes, especially for the non-motor domain; but, it has also obvious limitations, as it is neither able to give information about the underlying structure nor the direction, how different areas are influenced by each other. Due to its importance, we point to the section “Limitations of Functional rsMRI” (see above), which should not be neglected in the context of rs-fMRI network analyses.

One additional fact has to be mentioned: whereas MEG has the ability to measure activity changes in different networks with a high temporal resolution (but low spatial resolution), rs-fMRI shows changes in different brain networks with a high spatial resolution (as here exemplary described for the somato-motor, DMN, frontal-parietal, salience and visual network). However, despite this very high spatial resolution, it measures BOLD changes, which are the hemodynamic, indirect, answers to brain activity; hence the temporal resolution of rs-fMRI is low. Herewith the combination of rs-fMRI and MEG analyses seems reasonable to close the gap between temporal and spatial resolution in order to capture the full disease pathology. QSM imaging might herewith give an additional answer of the structural disease pathology underlying these functional changes (Wang et al., 2016).

Central to the pathophysiology of PD is the oxidative stress and a pathological protein aggregation, which leads to the degeneration of neurons (Dias et al., 2013; Hwang, 2013); specifically dopaminergic neurons are affected in PD. A number of mechanisms for the generation of reactive oxygen species exist, including the metabolism of dopamine itself, mitochondrial dysfunction, iron, neuro-inflammatory cells, calcium, and aging (Medeiros et al., 2016). QSM imaging enables the analysis of intrinsic tissue property with a mapping of iron (Haacke et al., 2015) and herewith QSM imaging delivers a new method to get basic information about the disease pathology in PD and should be integrated with studies about non-motor symptoms in PD. The accumulation of α-synuclein is a key neuro-pathological finding in parkinsonian brains. It is a small soluble protein and the primary structural component of Lewy bodies (Spillantini et al., 1997). Hence, Rietdijk et al. (2017) postulated a distribution of Lewy bodies throughout the whole brain, although the predictive value for disease stage of PD has been re-evaluated critically (Burke et al., 2008). It has been found that the interactions between α-synuclein and iron are closely related and the appearance of each other yields to a vicious circle, in which higher levels of α-synuclein evoke increased iron accumulation. This iron accumulation hence triggers the aggregation of α-synuclein (Lingor et al., 2017) and increases the oxidative stress with a destruction of neurons (Sian-Hülsmann et al., 2011); therefore QSM imaging, measuring the impaired iron distribution in the parkinsonian brain, might give the neuropathological answer to dysfunctional neuronal networks (measured by MEG and rs-fMRI) in PD.

Although QSM imaging has a strong impact on the imaging of subcortical levels (especially of the SN), the role of QSM at the cortical level for non-motor symptom studies is still questionable. In most of the QSM imaging studies using the regularization method [except for **C**alculation **o**f **s**usceptibility through **m**ultiple **o**rientation **s**ampling, COSMOS (Liu et al., 2015), which is not practical, but can obtain the gold-standard map through QSM imaging in various directions] discussions exists of how to overcome the artificial errors in cortical levels. Problems exist especially in overcoming the inverse problem of QSM imaging due to the missing discrimination of iron at cortical levels, their relations between QSM value and the aggregation of α-synuclein (Wang and Liu, 2015). The complex underlying physics raises the question “of how the signal is influenced in QSM imaging” (Acosta-Cabronero et al., 2016): (i) excessive background susceptibility effects, imperfect QSM inversion, registration errors; (ii) signal changes due to individual parameters (e.g., age, symptom onset); (iii) other paramagnetic metals (e.g., copper, manganese). All these factors require a careful handling and make the interpretation of whole-brain QSM imaging challenging. The new whole-brain approach of Acosta-Cabronero et al. (2017) should, from our point of view, be integrated in future QSM analyses to enable a pathophysiological interpretation of functional changes also in the non-motor domain. In line with this, the feasibility of integration of whole-brain QSM analyses in the non-motor domain has been recently shown for cognitive decline in patients with PD, showing that a higher iron load in the bilateral hippocampus is present in PDD patients (Li et al., 2018). The on-going development of new QSM imaging schemes (Jang et al., 2019) and higher field strengths in QSM imaging (e.g., 7T; Deistung et al., 2013) opens the additional possibility for *in vivo* histology and might herewith yield to a better comprehension of the development of parkinsonian phenotypes and their relationship to the iron metabolism.

## Conclusion

QSM imaging is a new method that is applicable to detect pathophysiological changes in the parkinsonian brain, which rely on iron accumulation indirectly interfering with the α-synuclein pathology. Together with functional measurements (like rsMRI and MEG) QSM imaging might open the possibility to better classify different parkinsonian phenotypes (including both the motor and non-motor domain) *via* a combination of structure and function. The further classification of parkinsonian phenotypes could provide insights into different disease patterns and courses, which might open the way to new therapeutic strategies to reduce parkinsonian symptoms.

## Author Contributions

EP: substantial contributions to the conception and design of the work. Additionally, substantial contribution to the acquisition, analysis and interpretation of data for the work. EF: critically revising the manuscript and providing additional information in the MEG section. AS: drafting the work and revising it critically for important intellectual content.

## Conflict of Interest Statement

The authors declare that the research was conducted in the absence of any commercial or financial relationships that could be construed as a potential conflict of interest.

## Supplementary Material

TABLE S1 Overview of key results for QSM imaging studies.

TABLE S2 Overview of key results for resting state analyses of MEG studies.

TABLE S3 Overview of key results in resting state analyses for fMRI studies at the example of the somato-motor network.

## Abbreviations

AI: Anterior insula
ALFF: Amplitude of low frequency fluctuation
AR: Akinetic-rigidic
BOLD: Blood oxygen level dependent signal
CD: Caudate nucleus
CEN: Central execution network
dACC: Dorsal anterior cingulate cortex
DLPFC: Dorsal lateral prefrontal cortex
DMN: Default mode network
FC: Functional connectivity
FOG: Freezing of gait
GCA: Granger causality analysis
GP: Pallidum
GRE: Gradient echo
Hz: Herz
ICB: Impulsive control behavior
ICA: Independent component analysis
LFP: Local field potential
MCI: Mild cognitive impairment
MEG: Magnetoencephalography
MRI: Magnetic resonance imaging
PD: Parkinson’s disease
PDD: Parkinson’s disease dementia
PPC: Posterior parietal cortex
PUT: Putamen
QSM: Quantitative susceptibility mapping
ReHo: Regional Homogeneity
RN: Red nucleus
ROI: Region of interest
rs-MRI: Resting state functional magnetic resonance imaging
SMA: Supplementary motor area
SN: Substantia nigra
SNc: Substantia nigra, compact part
SNr: Substantia nigra, reticular part
STN: Subthalamic nucleus
TD: Tremor-dominant
THA: Thalamus
UPDRS: Unified Parkinson disease rating Scale.
