# Supplementary material for: Quantitative Susceptibility Mapping and Resting State Network Analyses in Parkinsonian Phenotypes—A Systematic Review of the Literature
Source: Front Neural Circuits. 2019 Aug 6;13:50. doi: 10.3389/fncir.2019.00050 (PMC6691025; doi:10.3389/fncir.2019.00050)
Supplement: TABLE S1 — Overview of key results for QSM imaging studies. [file Table_1.DOCX]

| **QSM** |  | **Higher QSM values in:** | **Correlations** | **Additional notes** |
| --- | --- | --- | --- | --- |
| *Langkammer* et al*. , 2016* | ROI-analysis | SN, GP, Tha, RN | LEDD, total MDS-UPDRS, MDS-UPDRS-I and II) |  |
| *He* et al., *2015* | ROI-analysis | bilat. SN, RN contralat. to most affected limb | Disease duration, UPDRS-III |  |
| *Murakami* et al., *2015* | ROI-analysis | SN |  |  |
| *Barbosa* et al., *2015* | ROI-analysis | SN (compact part) |  |  |
| *Zhao* et al., *2017* | ROI-analysis | SN |  |  |
| *Azuma* et al., *2016* | ROI-analysis | SN (compact part) |  |  |
| *Du* et al., *2016* | ROI analysis, voxel midbrain analysis | SN | Disease duration, LEDD, UPDRS II |  |
| *Acosta-Cabronero* et al., *2017* | Global QSM measurement  ROI analysis | **Rostral pontine areas**  - pyramidal tracts  - pontine tegmental areas  - locus coerulus  - superior cerebellar peduncle  **Caudal mesencephalon**  - SN, compact part  - ventral tegmental area  **Midbrain tegmental areas**  - dorsal raphe  - oculomotor nuclei  **Cortical regions:**  - temporal paralimbic  - prefrontal  - occipito-parietal  - insular  - cerebellar |  | Decreased QSM values in:  Dentate nucleus |
| *Guan* et al. *2017* | ROI analysis | Early stage PD: SNpc  Late stage PD: SNpc, SNpr, RN, GP | SNpc, GP with Hoehn & Yahr  SNpc with UPDRS-III |  |
| *An* et al., *2018* | ROI analysis | SN   - Suptype   - akinetic-rigidic  - tremordominant  - mixed type   - Symptom severity   - mild  - advanced | SN -> Hoehn & Yahr, UPDRS, MADRS, HAMA | Mild affected: MADRS, HAMA  Advanced: Hoehn & Yahr, UPDRS-III, UPDRS total score, HAMA, MARDS  Akinetic-rigidic subgroup mainly affected:  -> correlation between iron content in SN and MADRS, HAMA only in akinetic-rigidic subtype |
| *Sjöström*, et al., *2017* | ROI analysis | RN and GP PSP > PD, MSA, HC  RN and GP MSA > PD  SN PD > HC |  |  |
| *Alkemade et al., 2017* |  |  |  | Improved visualisation of STN |
| *Liu et al., 2013* |  |  |  | Improved visualisation of STN |
| *Ide et al. , 2015* |  |  |  | Improved differentiation between medial and lat. GP |
