# Supplementary material for: Quantitative Susceptibility Mapping and Resting State Network Analyses in Parkinsonian Phenotypes—A Systematic Review of the Literature
Source: Front Neural Circuits. 2019 Aug 6;13:50. doi: 10.3389/fncir.2019.00050 (PMC6691025; doi:10.3389/fncir.2019.00050)
Supplement: TABLE S2 — Overview of key results for resting state analyses of MEG studies. [file Table_2.DOCX]

| **MEG** |  | **Increase** | **Loss /Decrease** | **Additional notes** |
| --- | --- | --- | --- | --- |
| *Bosboom et al. , 2006* | Spectral power analysis (incl. FFT)  **PD with and without dementia vs. HC** | Non-demented PD vs. HC:  - Theta-power: diffusely  - Beta-power: diffusely | Non-demented PD vs. HC:  - Gamma-power: central and parietal | Demented PD:  - Further slowing involving delta and alpha bands,  - Reduced eye-opening reactivity |
| Stoffers et al. 2007 | Synchronization likelihood method  **De novo PD vs. HC** | - Theta-power (not frontal)  - Alpha-1 power (not frontal) | - Gamma-power (not right occipital)  - Beta-power (not frontal) | - Slowing of background MEG activity compared to controls |
| Stoffers et al. 2008 | Synchronization likelihood method  **Drug-naïve vs. HC** | - Alpha-1 range |  | - Significantly increased coupling in theta, alpha 2 and beta band functional connectivity in advanced PD stages  - Alpha-1 coupling earliest sign for cognitive dysfunction (“tendency for preservation”) |
| Stoffers et al. 2008 | Synchronization likelihood method  **Drug-naïve vs. PD with DRT** |  |  | - OFF-> ON state elevates slowing in 4-30 Hz range  - Strong motor response -> decrease in local beta-band coupling |
| *Bosboom et al. , 2008* | Synchronization likelihood method  **Demented vs. Non-demented PD** |  | - Alpha-range (fronto-temporal) in PDD | PDD vs PD  - Intertemporal: Lower delta., theta and alpha1-band synchronisation  - Centro.parietal: Decreased gamma-band synchronisation  - Parieto-occipital: higher alpha-2 and beta band synchronisation |
| Gómez et al. ,2011 | Lempel–Ziv complexity (LZC) method  **PD vs. HC** |  |  | - PD show less complex activity in 10 cortical regions (frontal, central, temporal, parietal and occipital on the right and left hemisphere) |
| Pollok et al. 2012 | ROI analysis (S1/M1)  **De novo PD vs. HC** | - Synchronized oscillatory activity at beta frequency (13-30 Hz) |  | - At rest and during isometric contraction  - Reversal via dopamine therapy |
| Olde Dubbelink et al. 2013 | Spectral power analysis  **PD (longitudinal, 2 timepoints) vs HC** | -Theta-power | -Dominant peak frequency  -Alpha-1 and Alpha-2 power | - Decreasing cognitive performance: increase of delta and theta power, decrease of alpha 1, alpha 2 and gamma power  - Motor impairment: increased theta-power only |
| Olde Dubbelink et al. 2013 | Beamforming method with PLI  Seed based analysis  **PD (longitudinal, 2 timepoints) vs. HC** | Baseline:  - alpha 1 (temporal) | Baseline:  -delta band FC (parahippocampal, temporal)  4-y-follow up:   - alpha 1 FC (middle temporal gyrus) - alpha 2 FC 8parahippocampal, inferior temporal, middle temporal, precuneus) |  |
| *Ponsen et al. , 2013* | Beamforming method with PLI  **PD vs. PDD** | PDD > PD:   - delta-power - theta power   (parieto-occipital, fronto-parietal) | PDD < PD:   - alpha power - beta power   (parieto-temporo-occipital, frontal areas) | Lower mean PLI values in delta and alpha bands  (fronto-temporal, parieto-tempro-occipital) |
| *Olde Dubbelink et al. 2014* | Graph Theory  **PD vs HC (longitudinal)** |  |  | - Impaired local efficiency (i.e. local clustering of connections) and network decentralisation early features |
| *Heinrichs-Graham et al. 2014* | Zero-lag phase locking value  **Treated PD vs untreated PD vs HC** |  | Unmedicated PD: beta-oscillations (bilateral motor regions) | Unmedicated PD: higher synchronicity between left and right motor cortex in beta-frequency range  Medicated PD: Normalisation of beta-frequency and oscillation after dopamine application |
| *Boon et al. 2017* | Beamforming method with PLI  **PD (longitudinal, 3 timepoints) vs. HC**  **Cognitive Decline -> Dementia** | - Beta-band informational outflow (basal ganglia, fronto-temporal regions) | - Beta-band informational outflow (parieto-occipital regions) | Low occipital outflow correlated with poor cognitive performance |
| *Cao et al. 2015* | Power spectrum analysis  **STN-DBS-OFF vs. STN-DBS-ON** |  |  | DBS_OFF: slowing of resting state oscillatory activity  DBS-ON: Suppressing synchronisation of alpha rhythm in somato-motor region |
| *Oswal et al. 2016* | Beamforming  **MEG and LFP recordings** |  |  | DBS suppressed synchronized activity in STN at low beta (13-21 Hz) rather than high beta (21-30 Hz)  DBS suppresses coupling of STN to motor cortical regions across the entire beta frequency band |
