# Supplementary material for: Quantitative Susceptibility Mapping and Resting State Network Analyses in Parkinsonian Phenotypes—A Systematic Review of the Literature
Source: Front Neural Circuits. 2019 Aug 6;13:50. doi: 10.3389/fncir.2019.00050 (PMC6691025; doi:10.3389/fncir.2019.00050)
Supplement: TABLE S3 — Overview of key results in resting state analyses for fMRI studies at the example of the somato-motor network. [file Table_3.DOCX]

| **Rs-fMRI** |  | **Method** | **Increased FC** | **Decreased FC** | | **Additional notes** |
| --- | --- | --- | --- | --- | --- | --- |
| *Somato-motor network* | Wu et al., 2009 | Graph theory  **PD (ON/OFF) vs. HC**  **Finger tapping task -> ROI identification** | Left cerebellum  Left primary motor cortex  Left parietal cortex | SMA,  DLPFC  Left Putamen | | Normalisation after dopaminergic application  *PD patients ON vs. OFF dopaminergic medication* |
|  | Esposito et al.,2013 | ICA analysis and spectral analysis  **Drug naïve PD vs. PD (under dopamine) vs HC** |  | SMA | | Levodopa stimulated reduced signal fluctuations in SMA |
|  | Göttlich et al. ,2013 | fMRI and graph theory  **PD (under dopamine) vs HC** | SMA  Superior parietal cortex  Posterior cingulate gyrus  Supramarginal gyrus | - occipital lobe (cuneus and calcarine)  - Medial and middle orbitofrontal cortex | | *PD ON dopaminergic medication* |
|  | Wu et al., 2011 | Seed based rs-fMRI  Pre-SMA, bilateral M1  **PD vs. HC** | Pre-SMA with:  Right M1 | Pre-SMA with:  Left Putamen  Right Insula  Right premotor cortex  Left inferior parietal lobule | | Stronger connectivity in the M1 with its own local region  *PD after 12 h dopaminergic withdrawal* |
|  | Yu et al., 2013 | Seed based rs-fMRI  (Putamen, Caudate, SMA)  **PD vs. Healthy controls** | Putamen with SMA |  | | *PD after 12 h dopaminergic withdrawal* |
|  | Helmich et al., 2010 | Seed-based ROI analysis  Posterior putamen, anterior putamen, caudate nucleus  **PD vs. HC** | Anterior Putamen with:  - **parietal operculum**  - **supramarginal gyrus** (rostral part of the inferior parietal gyrus)  - **insula**  - inferior temporal gyrus | Posterior putamen with:  - cingulate motor area  - bilateral postcentral gyrus  - **parietal operculum**  - **supramarginal gyrus** (rostral part of the inferior parietal gyrus)  - precentral gyrus  - superior temporal gyrus  - **insula**  - fusiform gyrus | | In HC:  Posterior putamen-> coupled to cortical motor areas (SMA and M1)  Anterior Putamen -> pre-SMA and anterior cingulate gyrus  Caudate nucleus -> DLPFC  *PD after 12 h dopaminergic withdrawal* |
|  | Manza et al. 2015 | **Seed-based ROI analysis**  Anterior and posterior Putamen, ventral and posterior Caudate nucleus  **Drug-naïve PD, PD with and without cognitive decline and motor deficit** | Dorsal caudate with:  - rostral anterior cingulate cortex  **-> cognitive decline** | Anterior putamen with  - midbrain (including SN)  **-> motor deficit** | |  |
|  | Hacker et al., 2012 | **Seed-based ROI analysis**  Caudate nucleus, anterior and posterior putamen  **PD vs. HC** |  | Striatum with:  -Thalamus  - Midbrain  - Pons  - Cerebellum | | Focally altered FC in sensorimotor and visual areas, supramarginal gyrus  Grading of FC in the Striatum with the brainstem  (posterior putamen > anterior putamen > caudate nucleus) |
|  | Agosta et al. 2014 | **Seed-based ROI analysis**  Caudate, Putamen, Globus pallidum, Thalamus  **Drug-naïve PD vs treated PD vs HC** | **Drug-naïve PD vs. HC:**  Left with right basal ganglia  **Treated PD vs. HC and drug naïve PD**:  Striatum with  - temporal cortex  Thalamus with:  - sensorimotor regions  - parietal regions  - occipital regions | **Drug-naïve PD vs. HC:**  Caudate nucleus and thalamus with:  - ipsilateral frontal areas  - ipsilateral insular areas  **Treated PD vs. HC and drug-naïve PD**:  Striatum with  - thalamic regions | |  |
|  | Bell et al. 2015 | **Seed-based ROI analysis**  14 regions in the Striatum  **PD ON and OFF medication vs. HC** |  | **PD OFF Medication vs. HC**  Striatum with:  - Thalamus  - Sensori-motor networks | | Normalisation of functional decoupling after dopaminergic application |
|  | Kwak et al. 2010 | **Seed based ROI-analysis and frequency content analysis**  (1) inferior and (2) superior ventral striatum, (3) dorsal caudate, (4) dorsal caudal and (5) rostral and (6) ventral rostral putamen  **PD OFF/ ON medication vs. controls** | **PD OFF medication vs. controls:**  Basal ganglia oscillatory activity with thalamo-cortical networks |  | | Levodopa reduces the amplitude of low frequency oscillations in an otherwise functionally hyperconnected brain |
|  | Baudexel et al. 2011 | **Seed-based ROI analysis**  STN  **Early-stage PD OFF medication vs. HC**  **Tremor PD vs. Non-Tremor** | STN with:  - primary motor cortex (M1)  - premotor cortex  - SMA  M1 hand area with:  STN area  **Tremor PD vs. HC:**  STN with  - hand area of M1  - primary sensory cortex |  | |  |
|  | Kuurani et al. 2015 | **Seed-based ROI analysis**  Unilateral STN  **Drug-naïve vs. treated PD vs. HC** | **Drug-naïve and PD OFF medication:**  STN with:  Sensori-motor cortex |  | | Correlation with UPDRS-III |
|  | Fernández-Seara et al. 2015 | **Seed-based ROI analysis**  STN  **PD vs. HC** | STN with:  - left primary cortex  - secondary motor cortices |  | | Arterial spin labelled perfusion fMRI |
|  | Mathys et al. 2016 | **Seed-based ROI analysis**  STN  **PD under dopamine vs HC** | Negative STN coupling:   - bilateral intraparietal sulcus/superior parietal cortex - right sensorimotor - right premotor - left visual cortex | Negative STN coupling:  Crus I of right cerebellum  Right ventromedial prefrontal region  Positive STN coupling:  - left insula  **-> severity of motor symptoms** | | Decline of interhemispheric functional connectivity between the left and right STN  **-> progression of PD-related motor-symptoms** |
|  | Shen et al. 2017 | Seed-based ROI analysis  STN  **PD under dopamine vs. HC** | STN with:  Sensorimotor cortex  **-> motor symptom severity** | |  | Only PIGD subgroup (vs. tremor subgroup) showed increased FC between right STN and sensorimotor cortex under medication |
